# Evaluation of Immunological Parameters in Pregnant Women: Low Levels of B and NK Cells

**DOI:** 10.1055/s-0039-1683903

**Published:** 2019-03-25

**Authors:** Juliana Araújo de Carvalho Schettini, Thomás Virgílio Gomes, Claudeir Dias da Silva Júnior, Sandra de Andrade Heráclio, Isabela Cristina Coutinho de Albuquerque Neiva Coelho, Leuridan Cavalcante Torres

**Affiliations:** 1 Instituto de Medicina Integral Professor Fernando Figueira, Recife, PE, Brazil; 2 Universidade Católica de Pernambuco, Recife, PE, Brazil

**Keywords:** B-lymphocytes, NK cell, T-Lymphocytes, pregnancy, immunophenotyping, reference range, linfócitos B, células NK, linfócitos T, gravidez, imunofenotipagem, intervalo de referência

## Abstract

**Objective** To describe the immunological and hematological reference intervals of low-risk pregnant women.

**Methods** A cross-sectional retrospective database analysis of a basic and translational study analyzing the hematological evaluation blood counts and immunophenotyping of TCD3 + , TCD4 + , TCD8 + , B, and natural killer (NK) cells of the peripheral blood in 79 low-risk pregnant women and of 30 control women from the state of Pernambuco, Brazil, was performed.

**Results** No significant differences were detected between the hematological profiles of the 2^nd^ and 3^rd^ trimesters. Nevertheless, the median level of B cells decreased significantly in the 2^nd^ (174 × 10^3^ µL; *p* < 0.002) and 3^rd^ trimesters (160 × 10^3^ µL; *p* < 0.001), compared with the control group (296 × 10^3^ µL). Similarly, the median level of NK cells was lower in the 2^nd^ (134 × 10^3^ µL; *p* < 0.0004) and 3^rd^ trimesters (100 × 10^3^ µL, *p* < 0.0004), compared with the control group (183 × 10^3^ µL). In contrast, relative TCD4+ and TCD8+ levels increased in the 2^nd^ and 3^rd^ trimesters compared with the controls (TCD4 + : 2^nd^ trimester = 59%; *p* < 0.001; 3^rd^ trimester = 57%; *p* < 0.01; control = 50%; and TCD8 + : 2^nd^ trimester = 31%; *p* < 0.001; 3^rd^ trimester = 36%; *p* < 0.01; control = 24%).

**Conclusion** Low-risk pregnant women have ∼ 40% less B and NK cells in the peripheral blood, compared with non-pregnant women. These parameters may improve health assistance for mothers and contribute to define reference values for normal pregnancies.

## Abstract (pt)

**Resumo:**

**Objetivo** Descrever o intervalo de referência imunológico e hematológico de gestantes de baixo risco.

**Métodos** Realizou-se uma análise retrospectiva, de um estudo básico e translacional, analisando o perfil hematológico e a imunofenotipagem das células TCD3 + , TCD4 + , TCD8 + , B e natural killer (NK) do sangue periférico de 79 gestantes de baixo risco e de 30 mulheres (controles) do estado de Pernambuco, Brasil.

**Resultados** Não observamos diferenças significativas entre os perfis hematológicos do 2° e 3° trimestres. No entanto, houve redução das células B no 2° (média = 174 × 10^3^ µL; *p* < 0,002) e no 3° trimestres (160 × 10^3^ µL; *p* < 0,001), comparado com o grupo controle (296 × 10^3^ µL). A mediana das células NK foi menor no 2° (134 × 10^3^ µL; *p* < 0,0004) e no 3° trimestres (100 × 10^3^ µL; *p* < 0,0004), comparado com o grupo controle (183 × 10^3^ µL). Porém, o percentual de TCD4+ e de TCD8+ aumentou no 2° e 3° trimestres em relação aos controles (TCD4 + : 2° trimestre = 59%; *p* < 0,001; 3° trimestre = 57%; *p* < 0,01; controle = 50%; e TCD8 + : 2° trimestre = 31%; *p* < 0,001; 3° trimestre = 36*%;* *p* < 0,01; controle = 24%).

**Conclusão** Mulheres grávidas de baixo risco têm ∼ 40% menos células B e NK no sangue periférico em comparação com mulheres não grávidas. Estes parâmetros podem melhorar a assistência à saúde das mães e contribuir para a definição de valores de referência para gestações normais.

## Introduction

Pregnancy (and its outcome) is affected by hematological parameters that are influenced by environmental, genetic, and hormonal factors.^1^ ^2^ ^3^ Many hematological changes occur during pregnancy due to hormonal variation. The total blood volume (red and white blood cell [WBC] mass) increases, while platelet counts decrease, especially in the 3^rd^ trimester.^3^ ^4^ Alterations may occur in the maternal peripheral blood, in the composition of cellular subsets of the immune system and in the maternal-fetal interface at the endometrial uterine wall.^1^ ^3^

Invasion of allogenic fetal cells, vascularization (especially spiral arteries), and the formation of the placenta may cause inflammation at the maternal-fetal interface.^5^ ^6^ ^7^ Preventing a strong inflammatory response and ensuring maternal tolerance to the fetal semiallograft are essential for a normal pregnancy.^5^ ^6^ ^7^ In addition, the maternal immune system has to protect both the mother and the fetus from infections.^8^ ^9^ Also during pregnancy, some hormones, including progesterone, estradiol, and human chorionic gonadotropin, modulate the immune system, being therefore essential for the maintenance of the pregnancy.^10^ ^11^

Changes in the levels of several cell types (with different functional roles) during pregnancy have been well documented. These changes include the levels of TCD4+ cells and their effector subsets, such as Th1, Th2 and Th17, regulatory T cells (Treg), B cells, regulatory B cells (Breg) and natural killer (NK) cells, which together orchestrate the immune status of the system.^5^ ^6^ ^7^ ^8^ ^9^

There are several studies reporting the levels of T, B and NK cells during different pregnancy stages.^12^ ^13^ However, most studies report the immunological profile (levels of T, B and NK cells) in high-risk pregnancies, such as those in which the pregnant woman presents with preeclampsia or gestational diabetes.^14^ ^15^ Therefore, for these studies and for others that analyze the hematological and immunological profile of pregnant women, the reference values of cellular components of the immune system and hematological parameters of low-risk pregnant women are important in order to discriminate between healthy and unhealthy people and also to compare national and worldwide results.

The main purpose of the present study was to describe the immunological and hematological reference intervals during pregnancy. Therefore, we have performed a hematological evaluation (blood counts) and immunophenotyping of peripheral blood T cells (CD3 + , CD4 + , and CD8 + ), of B cells, and of NK cells in healthy low-risk pregnant women.

## Methods

This was a cross-sectional retrospective database analysis of a basic and translational study performed at an outpatient pregnancy clinic (OPC) and at the Laboratório de Pesquisa Translacional of the Instituto de Medicina Integral Professor Fernando Figueira (IMIP) in Recife, state of Pernambuco, Brazil, between August 2013 and June 2014. The IMIP is the largest reference hospital for pregnant women in the northeast region of Brazil. The established clinical and laboratory protocols were submitted and approved by the IMIP Research Ethics Committee (protocol number 3437-13) and by the National Council of Scientific and Technological Development (CNPq, in the Portuguese acronym) (CAAE = 12528213.8.0000.5201). Informed consent was signed by all of the participants.

## Study Population

We have evaluated 79 Brazilian mothers (average age: 28 years old; age range: 18–41 years old) recruited by the OPC-IMIP at the time of antenatal care. A total of 4 mL of maternal peripheral blood anticoagulated with ethylenediaminetetraacetic acid (EDTA) was collected for the automated blood count, which was performed with an Abbot Cell-Dyn 3700 hematology analyzer (Abbott Laboratories, Chicago, IL, USA). All of the participants were submitted to screening for the presence of human immunodeficiency virus (HIV), hepatitis B and C viruses, human T lymphotropic virus type 1 and 2, and to an antinuclear antibody test. None of the patients presented primary and/or secondary immunodeficiency. The inclusion criterion was low-risk single pregnancies in the 2^nd^ or 3^rd^ trimesters without any infectious diseases. None of the patients had clinical signs of infection during pregnancy and at the time of the peripheral blood collection. The exclusion criteria were (i) family or personal history of autoimmune diseases and/or positive antinuclear antibody test, (ii) infections, (iii) history of bone marrow or organ transplant, (iv) in vitro fertilization, (v) personal history of malignancy, and (vi) primary and/or secondary immunodeficiency. The controls were healthy fertile women with no signs of infection and who met the inclusion and exclusion criteria of the present study.

## Immunophenotyping of Total T, TCD4 + , TCD8 + , B, and Natural Killer Cells

**Fig. 1:**
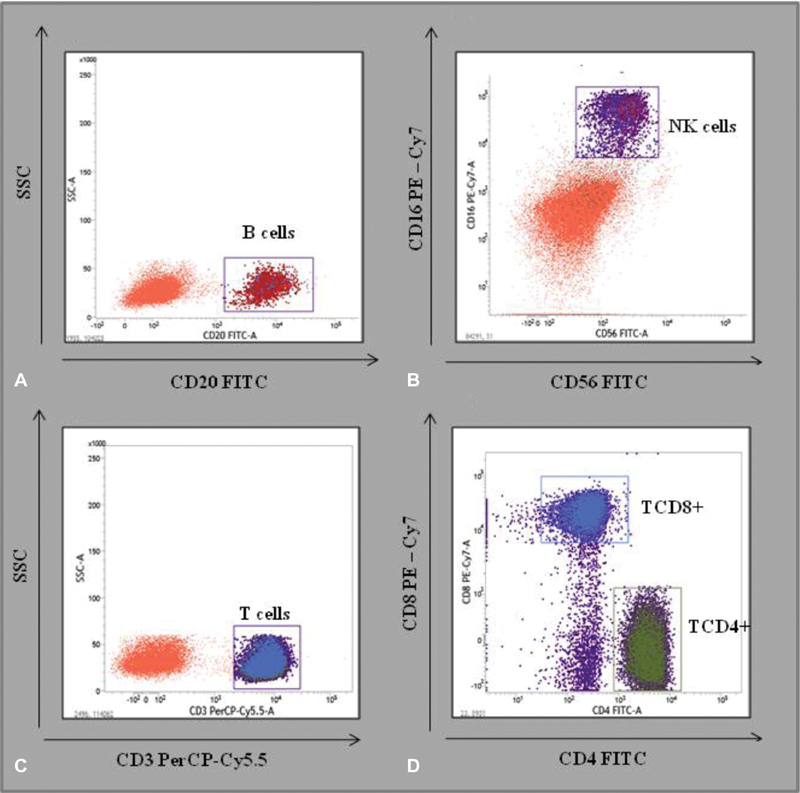
Representative density plots showing analyzes of: A) B cells (CD20+); B) NK cells (CD56+; CD16+); C) T cells (CD3+); D) T cells (CD4+ and CD8+).

Four-color flow cytometric immunophenotyping of the peripheral blood was performed on a BD FACSVerse cytometer (BD Biosciences, San Jose, CA, USA). The following monoclonal antibodies were used: anti-CD3, anti-CD4, anti-CD8, anti-CD16, anti-CD56, anti-CD19 and anti-CD20 (BD Biosciences, San Jose, CA, USA). The cells were analyzed using the most appropriate lymphocyte gate, with a combination of forward and side scatters (Fig. 1). The data obtained were analyzed using the BD FACSuite software (BD Biosciences, San Jose, CA, USA). The results were expressed as the percentage of positive cells within the selected gate. The mean fluorescence intensity index was used as measurement for the intensity of expression per cell.

## Statistical Analysis

The statistical analysis was performed using the Prism 6 software, (GraphPad Software, San Diego, CA, USA). Data are presented as median, minimum and maximum, quartiles/interquartile range (IQR) (25^th^–75^th^ percentile) and percentiles (10^th^ and 90^th^). The categorical variables are presented as percentage (%). The nonparametric Mann Whitney U-test was used to compare quantitative variables of two independent groups. Statistical significance was established at *p* < 0.05.

## Results

### Study Group

The study sample was composed of 79 Brazilian mothers. The median age of the pregnant women who participated in the present study was 28 years old (range: 18–41 years old). Most of the participants (*n = *50) (63.2%) were in their 2^nd^ trimester, 29 (36.7%) were in their 3^rd^ trimester, 45 (57%) carried a female fetus, and 34 (43%) carried a male fetus. Out of the total sample, ∼ 24% (19) were primiparous, 46.6% (37) were in the second pregnancy, 24% (23) had had 3 or more pregnancies, and 12.5% (10) had had a previous abortion. About 13% (10) of the pregnant women had completed secondary education, 47% (38) had completed primary education, 35% (27) had incomplete primary education, and 5% (4) had never studied. In the control group (non-pregnant women) the median age was 33 years old (range: 18–45 years old); 11% (3) had completed secondary education, 44% (13) had completed primary education, 40% (12) had incomplete primary education, and 5% (1) had never studied. The number of pregnancies among the controls varied from 1 to 5 (median: 2), the parity varied from 0 to 3 (median: 1), and 4 of the controls (13%) had had a previous spontaneous abortion. There was no statistically significant difference in height, weight, and body mass index (BMI) between the groups analyzed.

### Hematological Parameters

There was no statistical difference in the median values of red cell (RCC), hematocrit (Htc), hemoglobin (Hb), total leucocytes, monocytes, neutrophils, eosinophils, basophils, lymphocytes, and platelets counts between the 2^nd^ and 3^rd^ trimesters (*p* > 0.05) (Table 1).

**Table 1:** Hematological and Immunological parameters of Brazilian mothers in the 2^nd^ and the 3^rd^ trimesters of pregnancy

	2^nd^ trimester (*n *= 49)			3^rd^ trimester (*n *= 30)			
	Median	P10^th^–P90^th^	IQR	Median	P10^th^–P90^th^	IQR	*p*-value
RCC*	3,830	3,370–4,420	3,635–4,410	4,130	1,983–4,341	3,585–4,200	0.82
**Hct (%)**	35.3	30.7–39.5	33.1–37.9	35.7	28.22–39.89	32.43–37.98	0.42
**Hb (g/dl)**	11.8	10.5–13.1	11.1–12.6	12.1	9.28–13.31	11.13–12.75	0.69
Leucocytes**	9.4	6.1–13.9	7.4–10.5	9.4	5.3–12.3	7.2–10.55	0.39
Neutrophils**	6.6	4.3–9.7	5.4–8.2	6.5	3.7–8.7	4.9–8.1	0.39
Lymphocytes**	1.7	1.2–2.9	1.4–2.2	1.7	1.0–2.6	1.3–2.2	0.30
Monocytes**	0.5	0.2–0.8	0.4–0.6	0.6	0.27–0.84	0.39–0.72	0.33
Eosinophils**	0.01	0.09–0.33	0.11–0.25	0.01	0.06–0.33	0.09–0.16	0.22
Basophils**	0.02	0.010–0.063	0.01–0.04	0.02	0.045–0.087	0.02–0.04	0.95
Platelets**	232	145–318	196–291	216	152–359	182–267	0.06

Abbreviations: Hb: hemoglobin; Hct: hematocrit; IQR: interquartile range; RCC: red cell count.

* (×10^6^/mL); **(×10^3^/µl); Data are expressed as median, interquartile ranges (IQRs) and percentiles (P10^th^–P90^th^). *P* < 0.05 was considered statistically significant.

**Immunological parameters**: The immunophenotyping of Brazilian mothers during pregnancy is summarized in Table 2. There was no statistical difference between lymphocyte subsets of the 2^nd^ and 3^rd^ trimesters (*p* > 0.05) (Table 1). Low relative and absolute levels of B cells (*p* < 0.003 and *p* < 0.001 respectively) and low absolute levels of NK cells (*p* < 0.019) were observed. High relative levels of TCD4+ (*p* < 0.01) and of TCD8+ cells (*p* < 0.0004) were observed in the 2^nd^ and 3^rd^ trimesters, compared with the control group (Table 2).

**Table 2:** Lymphocytes subsets in pregnant women and in the Brazilian female adult healthy population

	2^nd^ trimester (*n *= 49)		3^rd^ trimester (*n *= 30)		Women control *(n = *30)		2^nd^ trimester x controls (*p-value*)		3^rd^ trimester x controls (*p*-value)	
Cell subsets	(%)	(×10^3^/µl)	(%)	(×10^3^/µl)	(%)	(×10^3^/µl)	(%)	(×10^3^/µl)	(%)	(×10^3^/µl)
**T CD3+** ^**Median**^ [IQR]* [P10–P90]**	76.50 [70.4–78.6] [66.5–83.2]	1,320 [980–1,502] [758–2,372]	76.30 [71.5–79.6] [58.2–83.2]	1,282 [830–1,593] [634–1,691]	73 70.5–77.5 70.1–81	1386 1164–1820 982–2160	0.38	0.30	0.34	0.10 0.10
**T CD4+** ^**Median**^ [IQR]* [P10–P90]**	59 [55–64] [50–69]	805 [619–1,043] [467–1,581]	57 [52–62] [48–67]	746 [537–877] [386–933]	50 42–55 38–64	902 645–1128 547–1326	0.0001*	0.63	0.01*	0.08
**T CD8+** ^**Median**^ [IQR]* [P10–P90]**	31 [27–39] [18–42]	433 [303–538] [248–930]	36 [30–39] [26–44]	425 [290–616.2] [207–717]	24 18–31.3 17–37	449 312–567 237–725	0.001*	0.88	0.0004*	0.94
**B cell** ^**Median**^ [IQR]* [P10–P90]**	10 [8.6–13.4] [5.1–16.4]	**174↓** [121–240] **[78–240]↓**	**9.5↓** [7.7–13.0] [5.0–16.0]	**160↓↓** [120–215] **[55–312]↓**	13 10–15 8–18	296 203–358 133–493	0.02*	0.002*	0.003*	0.001*
**NK cell** ^**Median**^ [IQR]* [P10–P90]**	7 [4.8–10.5] [3.8–13.8]	**134↓** [72–210] **[59–300]↓**	**6.8↓** [3.8–8.3] [2.4–19]	**100↓↓** [61–140] **[45–189]↓↓**	9.5 6–12 5–14	183 139–237 99–331	**0.04**	0.0004*	**0.02**	0.019*

Data are expressed as median; *IQR = interquartile range; **percentiles P10th–P90th; **↓=** low levels; *p* > 0.05 in the analysis between; ^##^ 2.5%–97.5% percentiles.

## Discussion

The reference values of cellular components of the immune system and of the hematological parameters of low-risk pregnant women is important for antenatal care to discriminate between healthy and unhealthy people, and also to compare national and worldwide results. The use of reference intervals derived from non-Brazilian populations could lead to the misinterpretation of the laboratory test results and may result in incorrect clinical care for the pregnant women. We have found low P10 levels of RCC, Hct, Hb, leucocytes, neutrophils, lymphocytes, and of eosinophils in the 3^rd^ trimester. However, based on the median levels, we did not find any statistically significant difference in the hematological parameters between pregnant women in the 2^nd^ and 3^rd^ trimesters. In contrast, Akinbami et al^2^ reported an increase in WBCs counts between the 1^st^ and 3 ^rd^ trimester of pregnancy in Nigerian women. Some authors have also observed a decrease in platelets during the 3^rd^ trimester.^2^ ^16^ Also, Genetu et al^16^ found higher Hct and Hg levels as pregnancy advances in an Ethiopian population. Ethnic peculiarities and some environmental factors are involved in these differences.

However, pregnant women in their 2^nd^ and 3^rd^ trimesters had low relative and absolute levels of B lymphocytes. As previously described in an animal model, this could be due to the migration of B cells to the human decidua and their differentiation into Breg cells. Some authors have demonstrated that interleukin 10 (IL10) produced by Breg cells can support the expansion of Treg cells.^17^ The latter may, in turn, participate in the development of maternal-fetal tolerance and also maintain dendritic cells in an immature state, inhibiting their capacity to present antigens and, consequently, to activate T cells.^17^ ^18^ Thus, low B cell counts in the peripheral circulation during pregnancy may be related to infiltration in the maternal-fetal interface, as described for animal models, affecting the maternal-fetal immune tolerance. Some authors reported that the number of conventional B cells remains relatively constant during human pregnancy. However, the maternal blood has lower proportions of B1 lymphocyte subsets during pregnancy, which return to nonpregnant women levels in the postpartum period. Levels of B2 cells remain unchanged in the maternal peripheral blood during pregnancy.^19^

Levels of NK cells decreased during the 2^nd^ and 3^rd^ trimesters of pregnancy compared with the control group. Contrasting the normal levels of NK cells in the peripheral blood, which account for a small fraction of the total lymphocytes (∼ 10%), NK cells are the dominant cell type in the decidua during normal pregnancies (∼ 50–90%).^20^ Interleukin 15 (IL-15), secreted by endometrial stromal cells, promotes migration of NK cells to the decidua. These cells have different functions: protection against infections (in the mother and in the fetus), angiogenesis, vascular remodeling (especially spiral arteries), and immune regulation.^20^ ^21^ There are two specific subpopulations of NK cells with distinct functions: cytotoxic or regulatory cells.

Cytotoxic NK cells are important determinants of recurrent pregnancy loss, adherent placenta accreta, rate of embryo implantation in in vitro fertilization, complicated pregnancies due to hypertensive disorders such as preeclampsia, and intrauterine growth restriction.^14^ ^21^ ^22^ ^23^ ^24^ Furthermore, high levels of NK cells in the peripheral maternal blood may be associated with adverse maternal and perinatal outcomes. Regulatory NK cells (CD56 bright/CD16-) are required for trophoblast control, for vascular remodeling in the regulation of tissue homeostasis, and to prevent strong inflammatory maternal responses.^5^ ^25^ ^26^

In addition, decidual regulatory NK cells promote maternal immune tolerance to the fetus by regulating inflammatory Th17 cells at the maternal-fetal interface.^6^ ^8^ ^25^ There is no consensus in the literature on the levels of TCD4+ and of TCD8+ cells in the peripheral blood during pregnancy.^5^ ^26^ ^27^ ^28^ However, high relative levels of TCD4+ and of TCD8+ cells in the peripheral blood of pregnant women are known to be important for the protection of the mother and of the fetus against bacterial and virus infections, caused for instance by arbovirus, adenovirus and HIV.^29^ ^30^ Furthermore, it has been suggested that the increase in TCD4+ and in TCD8+ cell levels compensates for the lower levels of B and NK cells in the peripheral blood. We could not evaluate longitudinally the selected parameters in the 1^st^, 2^nd^, and 3^rd^ trimester in all of the women and compare their concentrations among the trimesters because, unfortunately, there was a delay in the Brazilian public health system (SUS, in the Portuguese acronym) to confirm the antenatal consulting in the beginning of the pregnancy; however, this is a good objective for future research. We have found in the literature studies reporting the levels of T, B and NK cells during different pregnancy trimesters mainly in high-risk pregnancies, mostly from Europe and from the United States of America. However, the present study has been performed with low-risk pregnancies in South America. We have also analyzed the chemokines and cytokines of some pregnant women included in the population of the present study, and the data is free for consulting.^31^

Clinical and laboratory protocols were approved by the Research Ethics Committee of the IMIP (no. 3437-13). Written informed consent was obtained from all of the participants.

## Conclusion

In conclusion, low levels of total B and NK cells in the peripheral blood indicate that these cell populations are important for immune balance during pregnancy. Further research on the levels of the regulatory subsets of B and NK cells in the placenta tissue and in the peripheral blood may contribute to our understanding of immune tolerance during pregnancy.
